# The Maximum Entropy Method in Ultrasonic Non-Destructive Testing—Increasing the Resolution, Image Noise Reduction and Echo Acquisition Rate

**DOI:** 10.3390/e20080621

**Published:** 2018-08-20

**Authors:** Evgeny Gennadievich Bazulin

**Affiliations:** SPC ECHOPLUS, 123458 Moscow, Russia; bazulin@echoplus.ru; Tel.: +7-495-7809248

**Keywords:** antenna array, full matrix capture (FMC), triple scanning, structural noise, maximum entropy (ME), C–SAFT, total focusing method (TFM), time-of-flight diffraction (TOFD), code division multiple access (CDMA)

## Abstract

The use of linear methods, for example, the Combined Synthetic Aperture Focusing Technique (C–SAFT), does not allow one to obtain images with high resolution and low noise, especially structural noise in all cases. Non-linear methods should improve the quality of the reconstructed image. Several examples of the application of the maximum entropy (ME) method for ultrasonic echo processing in order to reconstruct the image of reflectors with Rayleigh super-resolution and a high signal-to-noise ratio are considered in the article. The use of the complex phase-shifted Barker code signal as a probe pulse and the compression of measured echoes by the ME method made it possible to increase the signal-to-noise ratio by more than 20 dB for the image of a flat-bottom hole with a diameter of 1 mm in a model experiment. A modification of the ME method for restoring the reflector image by the time-of-flight diffraction (TOFD) method is considered, taking into account the change of the echo signal shape, depending on the depth of the reflector. Using the ME method, 2.5D-images of models of dangling cracks in a pipeline with a diameter of 800 mm were obtained, which make it possible to determine their dimensions. In the object with structural noise, using the ME method, it was possible to increase the signal-to-noise ratio of the reflector image by more than 12 dB. To accelerate the acquisition of echoes in the dual scan mode, it is proposed to use code division multiple access (CDMA) technology based on simultaneous emission by all elements of the array of pseudo-orthogonal signals. The model experiment showed the effectiveness of applying the ME method.

## 1. Introduction

At present, in the practice of ultrasonic testing (UT), flaw detectors using antenna arrays are widely used. Two technologies are used to visualize the internal volume of objects: phased antenna array (PAA) technology [1], which is by far the most widely used technology and digital focusing with an antenna (DFA) technology [2,3]. In the paper [4] devoted to the comparison of the capabilities of PAA and DFA flaw detectors, it was concluded that DFA technology is more promising from an algorithmic point of view, and therefore the paper is devoted to the processing of echoes measured by DFA flaw detectors. One can afford such an analogy: PAA technology can be compared with dough, from which one can quickly bake only bread, and DFA technology is flour, salt and other ingredients that will allow one to knead the dough for any bakery or confectionery, but in this case, one need more time to get the final result.

**Figure 1:**
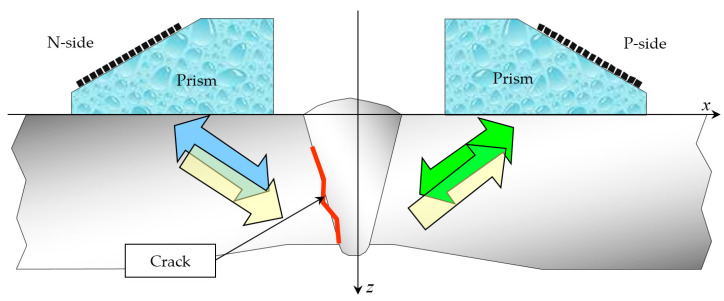
Scheme of measurements of echoes by two antenna arrays on prisms. Black rectangles schematically show the elements of the antenna array.

With single-sided access, typical for ultrasonic inspection practice, it is effective to use two antenna arrays of *N_e_* elements mounted on prisms and placed on the left (N–side) and on the right (P–side) from the welded joint to obtain a high-quality image of the reflectors. At the first stage of the application of the DFA-technology, echo signals are measured during the emission and reception by all combinations of pairs of elements of the antenna array [5]. In the paper [6], this mode of echo recording is called the dual scan mode, and in [7] the full matrix capture (FMC) mode. Echoes, radiated by one element and accepted by all elements, are called a shot. The equipment should support work on the N–(arrow with a blue fill in Figure 1), P–(arrow with a green fill) and NP–(arrows with a yellow fill) acoustic channels. The acquisition of echoes by an antenna array operating in the dual scan mode when it is mechanically moved is called a triple scan mode [6], or the migration of the antenna array [8]. Joint coherent processing of echoes by the C–SAFT method for different positions of the antenna array allows obtaining a high-quality image of the reflectors, which fundamentally distinguishes the algorithmic capabilities of DFA–flaw detectors from PAA-flaw detectors [4]. For echoes measured in triple-scan mode, it is possible to obtain focused images beyond the near-range of the antenna array, which is especially important for objects with a thickness of more than 70 mm and for objects with high structural noise.

In the second stage, the image of the reflectors $\varepsilon (r_{i})$ is reconstructed by the Combined Synthetic Aperture Focusing Technique (C–SAFT) [5,8], based on the measured echoes, which can be modified to take into account the multipath propagation of ultrasound in the object with uneven boundaries and the presence of regions with different acoustic properties [9,10]. Sometimes the C–SAFT algorithm is called the Total Focusing Method (TFM) [7]. In the Born approximation, the measured echo signals p can be represented as a system of linear Equations [7]:

(1) p=Gε+n,

where G is the matrix which describes the propagation of the ultrasonic pulse, n—the additive noise of the measurements. Note that the vector p and ε can be lexically formed from the matrices, and vice versa.

A fragment of the image having a maximum amplitude in the region of a sharp measurement of the acoustic properties of the monitoring object, for example, the boundary of the discontinuity, is called an indication. A false indication is an indication that does not correspond to the discontinuity boundary and is formed due to the limitations of the acoustic path model.

The higher the resolution of the reflector image and the lower the noise level, thus the number of reflectors, their sizes and type can be determined more accurately. This increases the reliability of the UT. The maximum entropy method, which is a nonlinear method, should increase the resolution and reduce the image noise level. The paper considers several options of the ME method application for obtaining high-quality image of reflectors and for increasing the speed of echo signal acquisition.

Section 2 summarizes the principles of reflectors image reconstruction from measured echoes. Section 3 describes a model experiment in which the possibility of increasing the signal-to-noise ratio of reflectors in materials with high attenuation when compressing complex signals by the ME method is demonstrated. In Section 4 a modification of the ME method for the case of a variable waveform measured in the TOFD mode is considered. Section 5.1 describes the results of a model experiment for obtaining a super-resolution image for a set of point-type reflectors and for a pipeline fragment of 800 mm diameter with fracture models. Section 5.2 presents the results of a model experiment to improve the image quality of flat-bottomed holes with a diameter of 1 mm. Section 5.3 demonstrates the effectiveness of the ME method to produce an image of side drilled holes in a composite weld with a high level of structural noise. Section 6 is devoted to improving the speed of echo acquisition in FMC mode using CDMA technology and the ME method.

## 2. ME Method in Ultrasonic Nondestructive Testing

### 2.1. Restoring the Reflectors Image

There are many ways to solve the classical problem by the Formula (1) from the point of view of solving the system of linear algebraic expressions. Because the matrix is typically ill-conditioned, in addition to a simple treatment of the matrix there are other solutions. One of them is to find an estimate as a solution of the unconditional optimization problem when the square of the solution residual is chosen as a criterion of the quality of the recovered image:

(2) $$\chi^{2}(\hat{\varepsilon })=\left\|G\hat{\varepsilon }-p\right\|={\left(G\hat{\varepsilon }-p\right)}^{T}(G\hat{\varepsilon }-p),$$

where the symbol *T* means the matrix transpose operation. The reconstructed estimate $\hat{\varepsilon }$ can be written as:

(3) $$\hat{\varepsilon }=\underset{\hat{\varepsilon }\in R^{N_{i,x}\cdot N_{i,z}}}{\arg\min}(\chi^{2}(\hat{\varepsilon })).$$

The solution of the inverse problem in the form (3) is called the least squares method. To search with the maximum speed of the minimum by Equation (3), one needs to use second order optimization methods [11]. Their application requires knowledge of gradient and Hessian, which for Equation (2) is calculated as follows:

(4) $$\nabla \chi^{2}(\varepsilon )=2G^{T}\left(G\varepsilon -p\right),\;\nabla \nabla \chi^{2}(\varepsilon )=2G^{T}G$$

In terminology [12], the solution of a degenerate system of linear algebraic Equation (1) with respect to providing the minimum of residual $\chi^{2}(\varepsilon )$ is called a pseudo-solution. There can be infinitely many pseudo-solutions and such parameters as resolution, speckle noise level, etc., may be far from ideal in general. Thus, in the work [13] the images of the reflectors based on the echo signals in the numerical experiment were found by the least square method. The image recovery algorithm was named extended synthetic aperture focusing technique (ESAFT).

There is an explicit regularized solution to Equation (3), which is presented below:

(5) $$\hat{\varepsilon }=\frac{G^{T}}{GG^{T}+\gamma E}p,$$

where E is the diagonal matrix, and γ is the spectral power density of the interference n. This formula is equivalent in structure to the formula that describes the Wiener filter in the frequency domain [14]. With the help of (5), it is possible to obtain high-quality images, but in the case of a low level of interference n.

Finally, the function $\hat{\varepsilon }$ estimate can be represented by analogy with the correlation formula in the matrix form:

(6) $${\hat{\varepsilon }}_{c}=G^{T}p,$$

To solve ill-conditioned problems a method of regularization was developed by Tikhonov [12], which justifies the replacement of the problem in the form of (1) by the problem of optimization of input data p resistant to small changes:

(7) $$\hat{\varepsilon }=\underset{\hat{\varepsilon }\in R^{N_{i,x}\cdot N_{i,z}}}{\arg\min}\left(\chi^{2}(\hat{\varepsilon })+\alpha \Omega (\hat{\varepsilon })\right),$$

where $\Omega (\hat{\varepsilon })$ is the stabilizing functional. The purpose of using stabilizing functionals is to take into account some *a priori* information about the solution when solving an ill-conditioned problem and thereby narrow the search for solutions. This *a priori* information can range from the simplest requirement for non-negativity of the solution, the minimality of the solution energy to restrictions on a known auto-correlation function, a given spectrum structure, and so on.

The entropy value of vector $\hat{\varepsilon }$ can be used as a stabilizing functional $\Omega (\hat{\varepsilon })$. The ME method is used in spectral estimation problems. In 1967, Berg [15] derived a formula to estimate the spectral density of the signal power by known samples of the autocorrelation function based on the requirement of the maximum entropy corresponding to the time series in the form $H(\varepsilon )=\sum \log\varepsilon_{i}$. Moreover, it was shown that the estimation of the spectral density of the signal power obtained by the autoregressive (AR) model and the spectral power density obtained by the maximum entropy method is identical in the case of the description of the signal as a Gaussian random process. Frieden in 1972 [16] showed the possibility of using Shannon entropy as a stabilizing functional in the context of the Tikhonov regularization method [12]. The possibility of achieving super-resolution in the image acquisition system (one-dimensional images, a system with diffraction limit) is demonstrated. Researches have shown the effectiveness of the practical application of the ME method in the reconstruction of images in tomography [17,18,19], radio astronomy [20,21,22], nuclear magnetic resonance [23], as well as in ultrasonic testing [24,25]. The paper [26] is devoted to the deconvolution of high-frequency (10–40 MHz) echoes by the maximum entropy method in the measurement of the thickness of layered structures.

Thus, the method of maximum entropy as a solution of the optimization problem with the entropy of image estimation as a stabilizing functional:

(8) $$\hat{\varepsilon }=\underset{\hat{\varepsilon }\in R^{N_{i,x}\cdot N_{i,z}}}{\arg\min}\left(\chi^{2}(\hat{\varepsilon })-\alpha H(\hat{\varepsilon })\right),$$

where H is the entropy of a set of discrete independent random variables, which is defined for the case of real, non-negative $\varepsilon_{i}$ as:

(9) $$H(\varepsilon )=-\sum_{i=1}^{N_{i,x}N_{i,z}}\varepsilon_{i}\ln\varepsilon_{i}=-\Omega (\varepsilon ),$$

where $N_{i,x}N_{i,z}$ is the number of pixels in the reconstructed image can be very useful for developing methods for obtaining image reflectors. The entropy calculated by this formula coincides with the entropy of a set of discrete independent random variables and is called Shannon entropy [27]. In practice, the so-called cross-entropy (other names: conditional entropy [27], relative entropy [20], the Kulbek-Labler distance is used [28]:

(10) $$H(\varepsilon )=-\sum_{i=1}^{N_{i,x}N_{i,z}}\varepsilon_{i}\ln\frac{\varepsilon_{i}}{m_{i}},$$

where m is an *a priori* model or evaluation of the type of solution ε. As a simple model, one can use a constant value eμ, in which μ is understood as an estimate of the average value of the background intensity of the image. The calculation of cross-entropy allows one to bypass one of the problems associated with the use of the maximum entropy criterion. The fact is that when some of the evaluation values ${\hat{\varepsilon }}_{i}$ approach zero, the logarithm in the expression for entropy (9) takes too large negative values, which makes it difficult to converge to images with zero background. For cross-entropy, its gradient elements will be close to zero for ${\hat{\varepsilon }}_{i}$ which are close to μ.

The logarithm in the maximum entropy criterion, as noted in [14], serves as an additional opportunity to prevent the appearance of elements with negative values in the recovered image. Since the recovered information ε about the reflector is considered as a real number, which can take both positive and negative values, Equation (10) cannot be used. Different approaches exist to adapt the maximum entropy criterion to data with negative values [29]. One can calculate the entropy of the module of vector ε in the form:

(11) $$\begin{array}{c} z_{i}=\left|\varepsilon_{i}\right|=\sqrt{{\varepsilon_{i}}^{2},}\\ H_{\left|\right|}(\varepsilon )=-\sum_{i=1}^{N_{i,x}N_{i,z}}z_{i}\ln\frac{z_{i}}{e\mu }. \end{array}$$

The gradient and the Hessian in the entropy calculation of Equation (11) will look as follows:

(12) $$\frac{\partial H_{\left|\right|}(\varepsilon )}{\partial \varepsilon_{i}}=-\frac{\varepsilon_{i}\ln\frac{z_{i}}{\mu }}{z_{i}},\;\frac{\partial^{2}H_{\left|\right|}(\varepsilon )}{\partial {\left(\varepsilon_{i}\right)}^{2}}=-\frac{\varepsilon_{i}}{z_{i}^{3}}.$$

In the article [30] it is proposed to calculate the entropy of the alternating function by the following formula:

(13) $$\begin{array}{c} z_{i}=\sqrt{{\varepsilon_{i}}^{2}-4\mu^{2}},\\ H_{\pm }(\varepsilon )=-\sum_{i=1}^{N_{i,x}N_{i,z}}\left(z_{i}-2\mu -\varepsilon_{i}\ln\frac{z_{i}+\varepsilon_{i}}{2\mu }\right). \end{array},$$

In this case, the gradient and the Hessian of the entropy will look as follows:

(14) $$\frac{\partial H_{\pm }(\varepsilon )}{\partial \varepsilon_{i}}=-\ln\left(\frac{z_{i}+\varepsilon_{i}}{2\mu }\right),\;\frac{\partial^{2}H_{\pm }(\varepsilon )}{\partial {\left(\varepsilon_{i}\right)}^{2}}=-\frac{1}{z_{i}}.$$

Considering that the entropy calculated by Equation (11) has two maxima, and the entropy calculated by Equation (13) has one maximum, Equations (13) and (14) were used in this paper. Note that in the Equations (12) and (14) the Hessian has the form of a diagonal matrix, which can significantly accelerate the work of optimizing algorithms and reduce the requirements for the sizes of the operative memory.

In the paper [31] for work with alternating sign functions the method of generalized entropy is proposed, when the alternating sign function ε is represented as the difference of two positive definite functions:

(15) $$\begin{array}{cc} \varepsilon_{+-}=\varepsilon_{+}-\varepsilon_{-},\;\mathrm{where}\; & \{\begin{array}{c} \varepsilon_{+}=\varepsilon_{+-},\;\mathrm{if}\;\varepsilon_{+-}\geq 0\\ \varepsilon_{-}=-\varepsilon_{+-},\;\mathrm{if}\;\varepsilon_{+-}<0 \end{array} \end{array},$$

This approach has a technical drawback. Since the size of the vector $\varepsilon_{+-}$ is doubled, the memory required for matrix G calculations is quadrupled.

### 2.2. Processing of Echo Signals Presented in a Complex Form

In the practice of ultrasonic testing, there are problems when it is necessary to increase the resolution of the pulses present in the measured echo signal p. In this case, we will mean by ε the type of the initial δ-pulses applied to the input of a linear system with a pulse response s. To solve the deconvolution problem, the matrix G columns will be formed by the response s with a cyclic shift, that is, the matrix G will be circulant.

In general, the vectors s, p and the matrix G can be complex. Similar to how it was done in [29], we introduce the following agreement: complex vector and the matrices will be written in the following extended form:

(16) p≡(pRepIm), G≡(GRe−GImGImGRe).

The gradient and Hessian of the residual criterion are calculated by Equation (4). The gradient and Hessian of the criterion H will be as follows:

H(ε)=−∑i=1Ntzilnzieμ, zi=(εiRe)2+(εiIm)2,∂H(εi)∂εiRe=−εiReln(zi/μ)zi, ∂H(εi)∂εiIm=−εiImln(zi/μ)zi,∂2H(εi)∂(εiRe)2=−εiRe−εiImln(zi/μ)zi3, ∂2H(εi)∂(εiIm)2=−εiIm−εiReln(zi/μ)zi3,∂2H(εi)∂εiRe∂εiIm=∂2H(εi)∂εiIm∂εiRe=εiImεiRe(ln(zi/μ)−1)zi3.

### 2.3. The Choice of the Lagrange Coefficient and Background Coefficient

The main methodological problem of practical application of the ME method is the choice of algorithm parameters: the evaluation of the background amplitude μ and the Lagrange coefficient *α*. The role of *α* is to establish a consensus between obtaining an exact solution of the ill-posed problem and satisfying the restriction caused by the stabilizing functional. There are precise and empirical methods for choosing the regularization parameter: the residual method [12,32], the cross-validation method [33], the generalized minimization problem [34], the L-curve method [35] and others. However, some methods are iterative and therefore require significant computational resources, while others require additional information, such as the noise dispersion value σ(n), which may not always be estimated with sufficient accuracy.

In many practical works devoted to the application of the ME method, the problem (7) is solved for the set of Lagrange coefficients α, and the best solution according to the given criterion is chosen from the set of obtained estimates ${\hat{\varepsilon }}_{\alpha }$. In this paper, from the set of estimates ${\hat{\varepsilon }}_{\alpha }$ for the set $\left\{\alpha_{1},\alpha_{2},\ldots ,\alpha_{N_{\alpha }}\right\}$, the estimate was chosen according to the L-curve criterion.

The introduction of another parameter μ and the need for its evaluation creates the additional complexity of the entropy calculation by the Formula (10). Researches have shown that when model and experimental data are restored, sufficiently close results $\hat{\varepsilon }$ are observed for the values of the background coefficient μ differing by several orders. In [30], it is recommended to choose a value μ equal to one-hundredth of the mean square value of the reconstructed image $\hat{\varepsilon }$.

## 3. Increasing the Signal-To-Noise Ratio of the Image Due to Deconvolution of Complex Signals

To increase the signal/noise ratio in objects with high absorption, complex signals can be emitted [36] if the radiating electronics of the flaw detector support this mode. After the compression procedure, which can be performed using matched filtering, the longitudinal resolution of the image will be about the same as using simple signals of minimal duration, but the signal ratio increases many times over. The compression of the echo signals using a matched filter can result in “side lobes”. In the paper [37], it was proposed to use the ME method for the compression of complex signals to achieve the effect of superresolution. Practical application of the ME method for compression of signals is impossible without a demonstration of its stability to the influence of noise.

**Figure 2:**
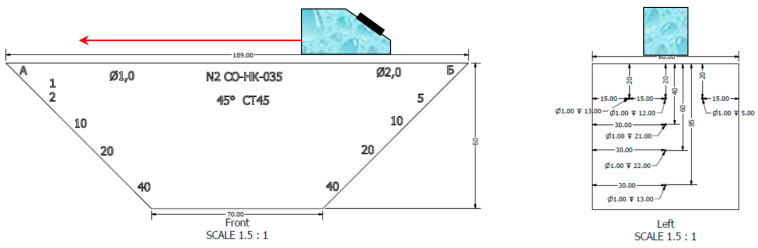
Drawing of the reference block with planar holes.

**Figure 3:**
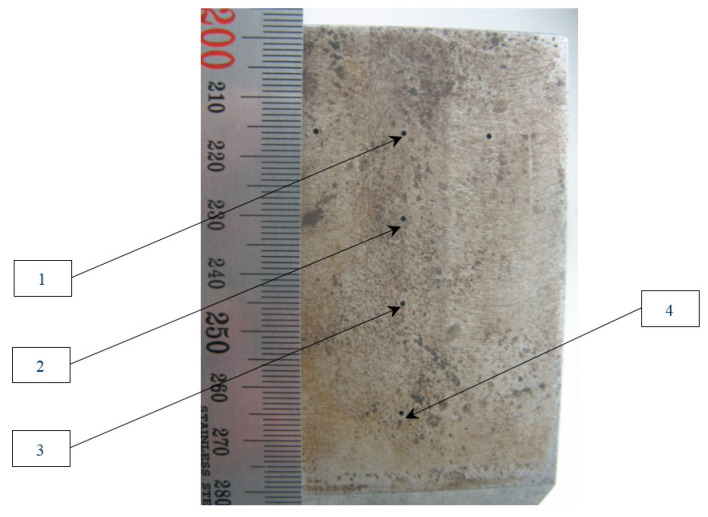
View of the reference block from the side of the holes with a diameter of 1 mm. The depth of flat-bottomed holes with numbers from 1 to 4 following 2, 10, 20 and 40 mm.

In the model experiment, the piezoelectric transducer (resonance frequency of 4.0 MHz, input angle of 40 degrees, half the opening angle of 20 degrees) moved along the scanning line, which is shown by the red arrow at Figure 2. Echo signals reflected from a 1.0 mm diameter flat-bottomed hole with a 45-degree slope at a depth of 40.0 mm were recorded (Figure 2 and Figure 3). The phase-manipulated signal according to the Barker code with a length of 13 was used as a probing signal.

**Figure 4:**
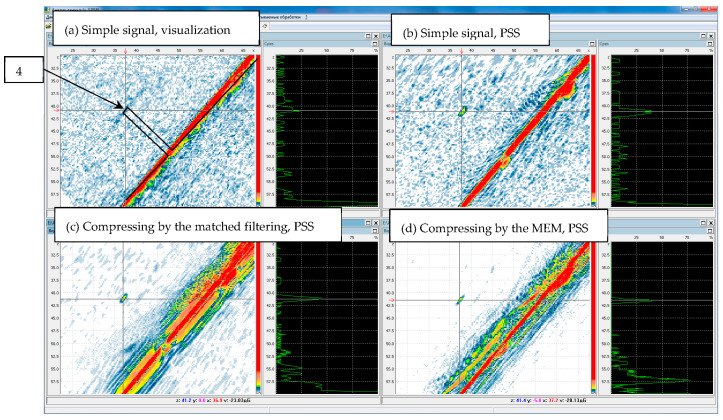
Images of the flat-bottomed hole 4, recovered from the original echoes (**a**), by the PSS method (**b**) and after their compression by the matched filtering (**c**) and by the ME method (**d**).

Figure 4a shows the result of visualization of the image of a flat-bottomed hole when a simple pulse is emitted. Visualization means finding the echo envelope and displaying it without spatial processing along the central beam of the transducer for all points of the spatial aperture. On the picture, the lines of black outline the boundaries of the hole and the sample. It can be seen that the indication from the hole, the center of which is indicated by a cross-shaped marker, is barely detected because of the noise level comparable with its amplitude. Figure 4b shows the result of the reconstruction of the image by these echo signals by the method of projection in spectral space (PSS or FT–SAFT) [38]. The signal-to-noise image obtained by the PSS increased by more than 12 dB in comparison with the visualization, and the front resolution decreased from 15 mm to 1 mm. Figure 4c shows the image of the bottom of the hole recovered by the method of PSS in the signals, compressed by the matched filtering, and Figure 4d—signals, compressed by the method of ME according to the Formula (8). The use of a complex signal resulted in an additional increase in the SNR of more than 12 dB. After the compression of the complex signal by the ME method, the noise level decreased by at least 20 dB, and the beam resolution decreased from 0.7 mm to 0.2 mm. Since the Barker code was used, the level of «side lobes» is insignificant after the compression of the echo signals by the matched filtration.

Thus, the use of complex signals and their compression by the ME method can increase the signal-to-noise ratio by more than 40 dB. This is a very large margin of sensitivity for ultrasonic testing in materials with high absorption.

## 4. Reconstruction of the Image by Tofd-Echoes

**Figure 5:**
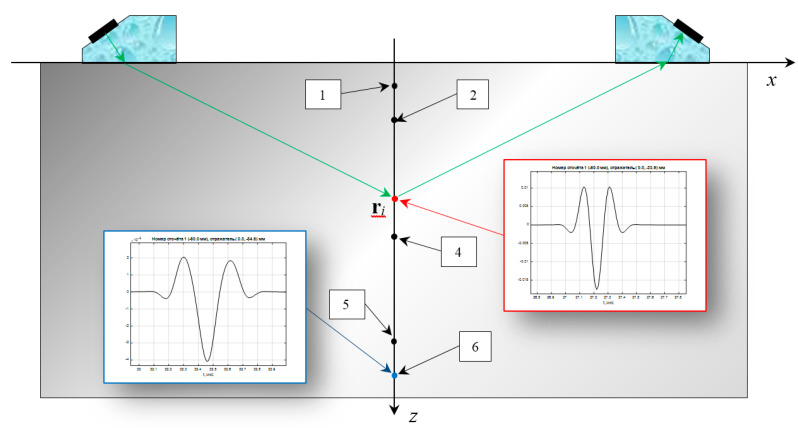
Echoes measurement in the TOFD mode.

In the practice of ultrasonic nondestructive testing, the time-of-flight diffraction (TOFD) [39] method is widely used, when pulsing and receiving transducers with a wide beam spread pattern move along the welded joint along an axis y perpendicular to the plane of Figure 5. The measured echo signals p(t,y) are informative and allow to detect cracks quite effectively, since the diffraction echo signals from the upper and lower edges of the crack differ in phase by about 180 degrees. The small size of acquired echo signals data set allows monitoring at a very high speed.

In Figure 5, the green arrows schematically show the trajectory of the propagation of the longitudinal wave pulse from the fragment of the radiating piezo crystal to the fragment of the receiving piezo crystal. The point-type reflector at the point $r_{i}$ is shown in red $\varepsilon (r_{i})=\delta (r-r_{i})$. To calculate the received echo signal, it is necessary to calculate the delay time and amplitude for all the rays when the pulse is emitted when an electric pulse $s_{e}(t)$ with a central frequency of 5 MHz is applied to the piezo crystal and to integrate on the surface of the piezo crystal, which will allow calculating the pulse $s_{trm}(r_{i},t)$ at the point $r_{i}$. A set of trajectories from the reflector to the surface of the receiving piezo crystal can be used to estimate the echo signal $p(r_{i},t)$ measured by the receiver. The finite dimensions of the piezo crystal lead to the fact that the shape of the measured echo signal will change. The pulse from the reflector (depth 25 mm) at the intersection of the acoustic axes of the transducers practically the same as the probe pulse and is shown in Figure 5 by the balloon with the red border. The echo signal from the point-type reflector at a depth of 45 mm is shown in Figure 5 by the blue balloon. The scale of both graphs is the same along the time axis and is equal to 1 µs, and the amplitudes are equalized. It can be seen that when the distance from the acoustic axis of the echo shape changes significantly.

Thus, after calculating $p(r_{i},t)$, it is possible to construct a matrix G and to reduce the problem of estimation ${\hat{\varepsilon }}_{MME}(z_{i})$ along the axis z by the measured echo signal p using the Equations (8) and (10). If the propagation time of the pulse is calculated from the centers of the of piezo crystal $t_{del}(z_{i})$ as a function of the depth of the point reflector, then the form ε(z) can be estimated from the formula ${\hat{\varepsilon }}_{del}(z_{i})=p(t_{del}(z_{i}))$. This formula can be considered as a degenerate version of the C–SAFT method.

**Figure 6:**
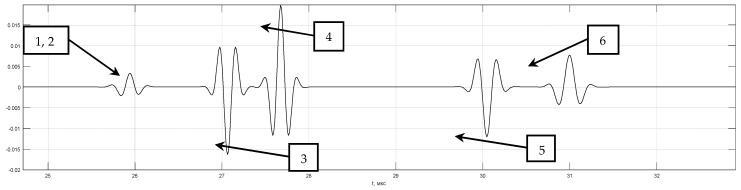
Echoes measurement in the TOFD mode.

Figure 6 shows the calculated echo signal p(t) from the six point-type reflectors for a distance between the front faces of the transducers is 120 mm. The first pair of reflectors with coordinates 2.5 mm and 7.5 mm, the reflection coefficients 1 and −1 simulates a crack with a height of 5 mm, similarly, the second pair has coordinates 22.5 and 27.5 mm, and the third one 42.5 and 47.5 mm. The pulse of the head wave and one reflected from the object bottom of the sample were not calculated. Echoes from the second and third pairs are resolved and have a phase shift of 180 degrees. Echo signals from the first pair of reflectors will not be resolved, so at low depths $t_{del}(z_{i})$ changes weakly. The amplitude and shape of pulses from the reflectors are different due to the divergence of the ultrasonic beam and the finite size of the piezoelectric plates.

**Figure 7:**
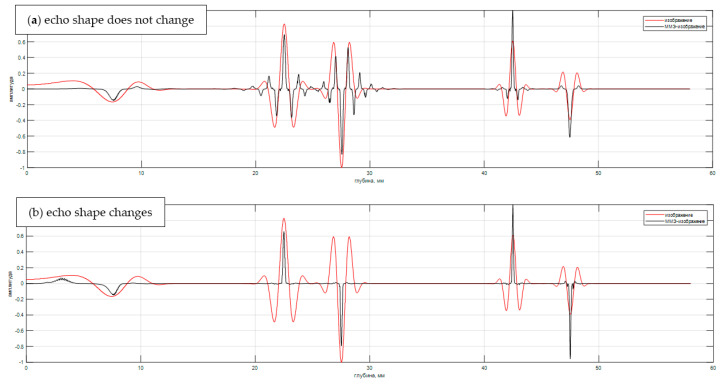
Image evaluation of reflectors e (z) and e (z) by TOFD–echoes.

Figure 7a shows the result of the evaluation of ${\hat{\varepsilon }}_{MME}(z_{i})$ (black graph) and ${\hat{\varepsilon }}_{del}(z_{i})$ (red graph) when the matrix G is formed taking into account the echo shape $p(z_{i},t)$ calculated for the depth of 45 mm. This led to the fact that the images of the third pair (reflectors 5 and 6) have the form of a δ–function, and the image of the reflectors of the second pair has «side lobes» of the order of 40%. Under ideal reconstruction of the values of the reflection coefficient $\hat{\varepsilon }(z_{i})$ in this example must be equal to 1 or −1. Figure 7b shows the result of the evaluation of ${\hat{\varepsilon }}_{MME}(z_{i})$ and ${\hat{\varepsilon }}_{del}(z_{i})$ at the calculation of the matrix G of echo shape $p(z_{i},t)$ for all depths. The ${\hat{\varepsilon }}_{del}(z_{i})$ has no difference from the shape *p*(*t*) except for the nonlinear scale transformation according to the dependency $t_{del}(z_{i})$, but the ${\hat{\varepsilon }}_{MME}(z_{i})$ has a resolution of more than five times higher than for ${\hat{\varepsilon }}_{del}(z_{i})$, which allows one to resolve reflectors 1 and 2. The amplitude of the reflector is 1 small since the calculation of $p(z_{i},t)$ did not take into account the feature of calculating the refractive index near the first critical angle [40]. It is clear that such a convincing result (Figure 7b) obtained in the absence of measurement noise and operator noise. To obtain better images of reflectors 1 and 2, it is necessary to bring together the emitting and receiving converters.

## 5. Improving the Image Quality of the Reflectors Reconstructed by the Me Method on a Set of Echoes

This section provides examples of the use of the ME method to restore the reflectors image from echo signals with super-resolution and increased signal/noise ratio (see section Restoring the image of reflectors).

### 5.1. Increasing the Resolution of an Image

#### 5.1.1. Four Side Drilled Holes

**Figure 8:**
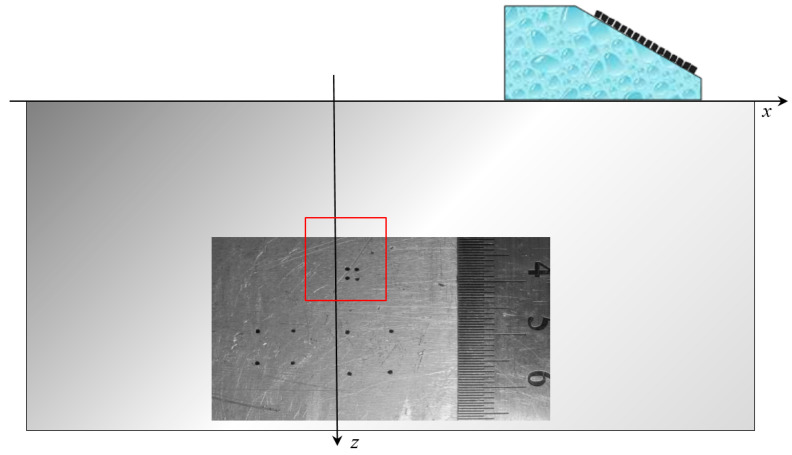
Rescattering test consists from three groups of four holes of 0.5 mm diameter.

Figure 8 shows a sketch of the duralumin block with 0.5 mm diameter side drilled holes. Image of holes located at a depth of 36 and 38 mm with a distance between centers about 2 mm was restored. For the acquisition of the echo signals of the used antenna array (5 MHz, 32 elements, the size of the piezo crystal is 0.9 × 10 mm, pitch 0.1 mm) on a plexiglass prism with a tilt angle of 20 degrees. The antenna array and prism are schematically shown in Figure 8.

**Figure 9:**
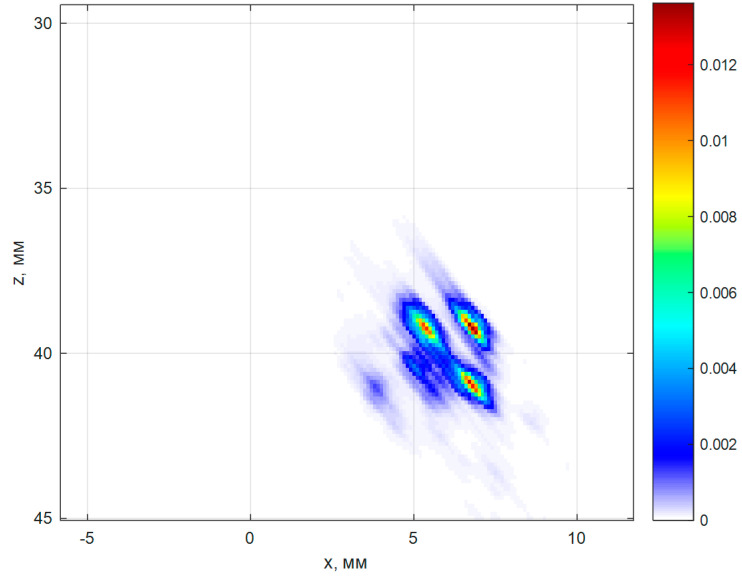
The image of the group of the side drilled holes, closest to the surface, of the rescattering test, reconstructed by the ME method.

Figure 9 shows the result of the reconstruction of reflectors by the C–SAFT method using 1024 echoes $p_{nm}(t)$. Non-sufficient resolution of the image does not allow to determine not only the type of the reflector but also their quantity. On the image lines of black color are plotted holes’ contour.

**Figure 10:**
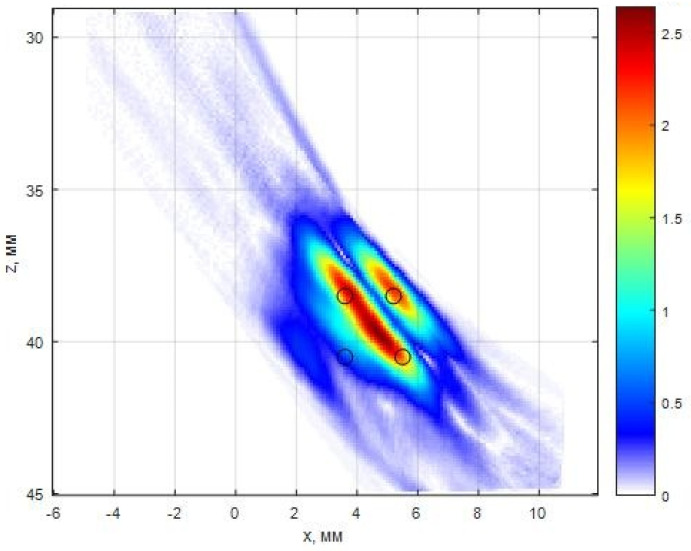
The image of the group of the side drilled holes, closest to the surface, of the rescattering test, reconstructed by the C–SAFT method.

Figure 9 shows the result of the reconstruction of the reflectors image by 400 echoes $p_{nm}(t)$ by the ME method (α=10.0, $\mu =10^{-5}$) according to Equation (8). The longitudinal and frontal resolution of the image increased more than twofold in comparison with the image of Figure 10. The image of the three holes is clearly seen in the ME image. The amplitude of the indication of the fourth hole was not large enough to detect it because of the shading effect.

#### 5.1.2. 2.5d–Image of Models of Two Cracks in Welded Joint of du800 Type

Model experiments were carried out with a welded joint of 800 mm diameter, in which seven artificial reflectors were made. This type of pipelines is typical for the nuclear power industry of the Russian Federation. Seven artificial reflectors were made in the weld. Reflectors with numbers 3 and 4 simulating the cracks have tilt 20 and −14 degrees to the axis z, a height of about 5 mm and the length along the weld about 34 mm. Reflector are milled grooves with a width of 1 mm. 

**Figure 11:**
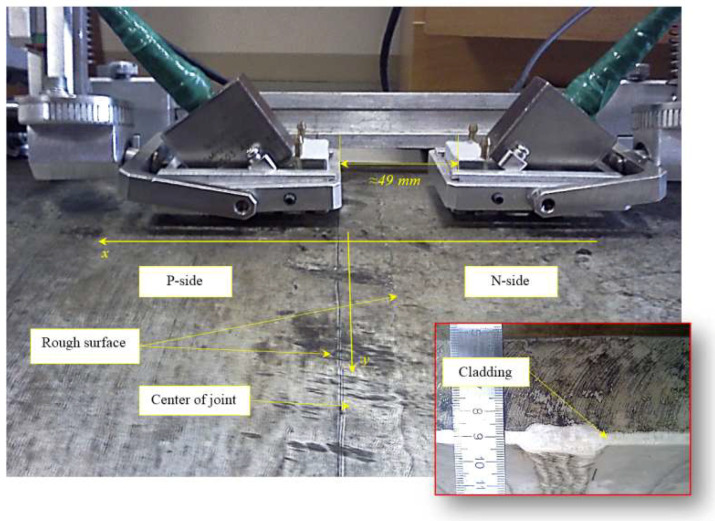
The weld from 800 mm diameter pipeline and two antenna arrays installed on the prisms are located approximately at the location of the grooves 3 and 4. The balloon shows the image of the end of the cutted weld.

Figure 11 shows a picture of the weld, part of the scanning system and two antenna arrays installed on the prism and located approximately at the location of the reflectors 3 and 4. The reinforcement bead of the weld on the outer surface has been removed. The antenna array (5 MHz, 32 elements, piezo crystal size 0.9 × 10 mm, pitch 0.1 mm) on a rexolite prism with a 35-degree angle of inclination was used to acquire the echo signals.

**Figure 12:**
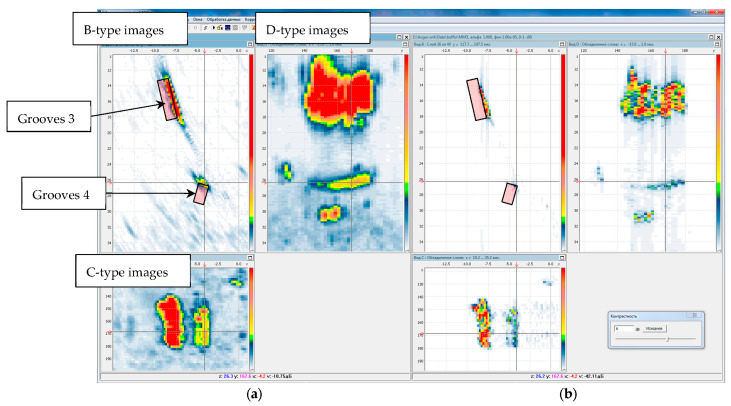
C–SAFT images (**a**) and ME images (**b**) for the P-side for the TdT acoustic scheme (a transverse wave is emitted, a transverse wave, reflected from the defect, is received). The D- and C-type images are combined to the maximum.

Figure 12 on the left shows the C–SAFT image, and on the right the ME image reconstructed according to Equation (8) obtained by the P-side antenna array. The B-type images show the contours of the grooves 3 and 4. It can be seen that the coordinates of the indications of the reflector 4 differ from the contours of the grooves. This can be explained by the fact that, firstly, the weld surface is rough. And, secondly, the welded joint can introduce additional distortions due to its special acoustic properties. The resolution of the ME image in comparison with the C–SAFT image has more than doubled. According to the D-type image, it can be assumed that the upper boundary of the groove 4 is rough, despite the fact that it is milled.

### 5.2. An Increase in the Signal-To-Noise Ratio of the Image for the Case of Large Absorption

In Section 3, when using a complex signal, it was possible to increase the image signal-to-noise ratio by more than 20 dB for a 1 mm diameter planar hole at 40 mm depth by deconvolution of each echo signal. Reconstruction of the image by the method of ME from a set of echoes by Equation (8) also allows one to increase its quality. To acquire echoes by FMC, a 16-element antenna array with an operating frequency of 4 MHz and a step of 2.5 mm was used, each element of which was mounted on an individual prism with an angle of 45 degrees.

**Figure 13:**
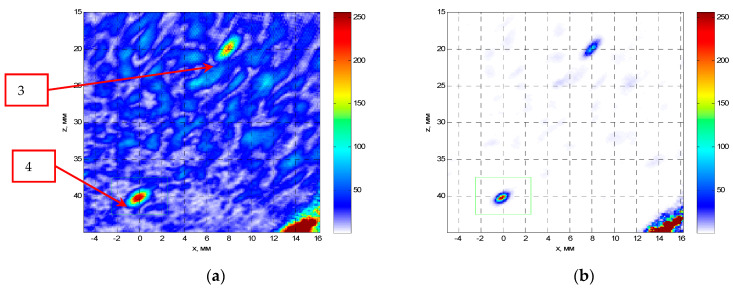
SAFT-(**a**) and ME-images (**b**) for the TdT acoustic scheme.

Figure 13a shows the image obtained by the correlation method according to Equation (6). The image is characterized by a high level of noise, where is not particularly clearly visible indications of the flat-bottom holes. Signal to noise ratio in the ME image reconstructed according to Equation (8) (Figure 13b), compared with Figure 13a, increased by 16 dB, when measured within the area marked with a green square, and the resolution increased by at least by three times.

### 5.3. An Increase in the Signal-To-Noise Ratio in Materials with Structural Noise

There are materials in which so-called structural noise occurs at the boundaries of crystal grains due to multiple rescattering of the probing pulses [41]. For such materials, it is not possible to increase the signal-to-noise ratio due to the increase in the energy input into the material, since simultaneously with the increase in the amplitude of the pulse reflected from the defect, increases the amplitude of the pulses reflected from the boundaries of the crystal grains. In coarse-grained materials, the main factor affecting the size of the minimum detectable reflector is not a small sensitivity, but a high level of structural noise. To reduce its level, it is recommended to measure using very short pulses [42]. The reconstruction of the image of the reflectors by the ME method should increase the resolution, which is similar to the reduction of the echoes length and this should lead to a decrease in the level of structural noise.

**Figure 14:**
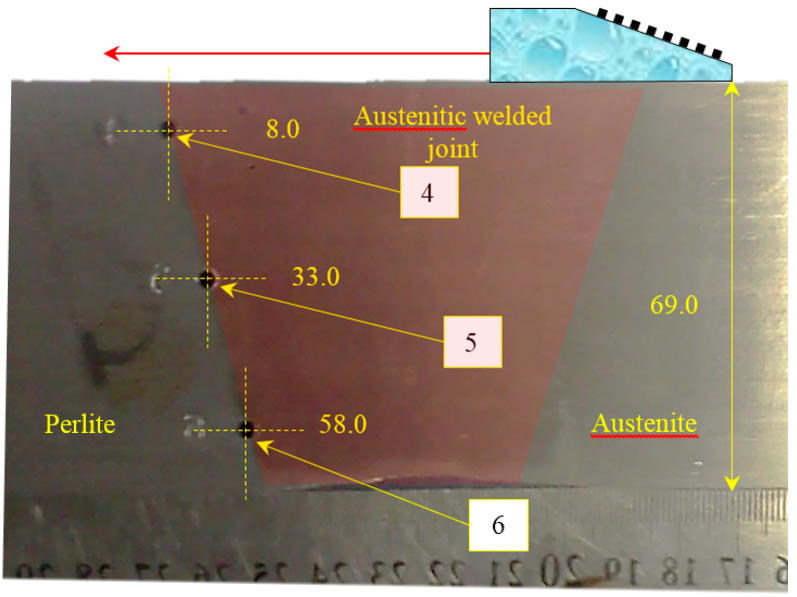
A view of a 69 mm pipeline sample with three side drilled holes.

Let us demonstrate this by the example of austenitic weld joint testing. The sample on which the measurements were carried out was a fragment of the pipeline with a thickness of 69 mm, with an austenitic welded joint (Figure 14). From one edge on the border of the “weld seam-base metal (perlite)” at depths of 8, 33 and 58 mm three 3.0 mm diameter side drilled holes were made, indicated by numbers 4, 5 and 6. The weld area is marked with a translucent red polygon. The longitudinal wave velocity in the weld joint was set to 5.6 mm/µs. The measurements were carried out in the mode of triple scanning at five points with a step of 9.3 mm antenna array (5 MHz, 32 elements, piezocrystal size 0.9 × 10 mm, 0.1 mm pitch) on a plexiglass prism with a 20-degree tilt angle. The scanning range is schematically shown in Figure 14 with a red arrow. One LdL acoustic scheme (the longitudinal wave is emitted, the longitudinal wave, reflected from the defect, is received) was used to reconstruct the images of reflectors.

**Figure 15:**
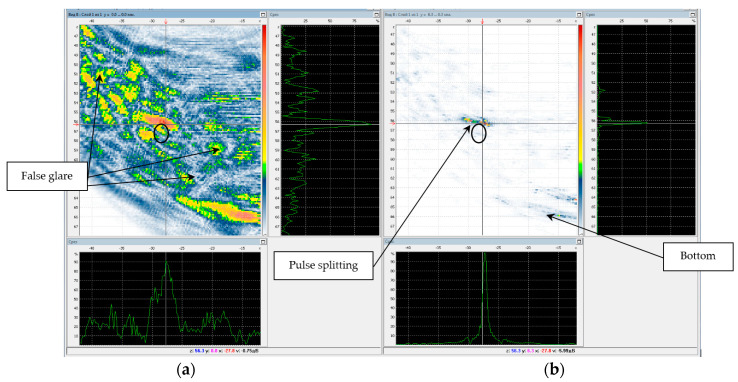
SAFT-(**a**) and ME-images (**b**) for the P-side for the LdL acoustic scheme.

Figure 15a shows the C–SAFT image of the boundary of the hole 6. The structural noise has a level of about 6 dB, against which one can see the indication of the hole, the contours of which are shown as black ellipse. Figure 15b presents the result of processing the same signals by the ME method according to Equation (8) after 15 iterations at the background coefficient $\mu =10^{-6}$. In comparison with the image obtained by the correlation method, the beam resolution of the image obtained by the ME method increased approximately three times, and the signal/noise ratio increased by more than 30 dB. However, the indication of the hole boundary on the ME image is split, due to the fact that when calculating the matrix G it is necessary to take into account different acoustic properties of the base metal and the weld joint.

## 6. Increasing of Echoes Acquisition Rate

The disadvantage of acquiring echoes by double scanning is a large amount of measured data. With an increase in the number of elements $N_{e}$ of the antenna array, the number of echoes increases as a square. Therefore, in spite of the fact that the echoes are measured in $N_{e}$ cycles of the probing signal, the transfer of a large volume of echoes from the measurement unit to the computer for image reconstruction may take a long time. 

**Figure 16:**
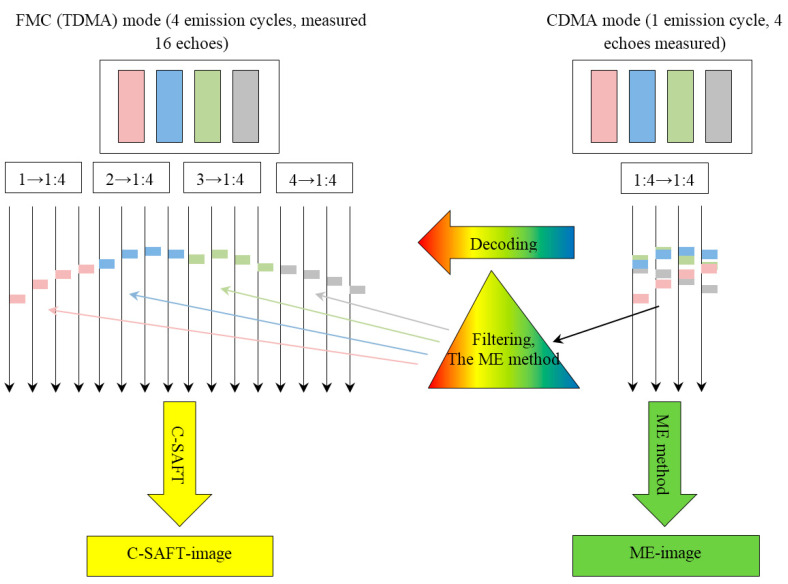
The principle of increasing the speed of recording echo signals.

From the point of view of the theory of multichannel communication, the dual scan mode is similar to the situation where users send a message in turn which is received by all users. The consistent nature of the radiation of the probe pulse allows us to answer the question: “Who is the source of the message?” This communication mode, when each pair of receiver-transmitter is allocated the whole spectrum or most of it for a selected period of time, is called time-division multiple access (TDMA) [43]. If all users could send their messages at the same time, and at the reception, it would be possible to select all the messages and understand: “Who sent the message?”—it would essentially increase the rate of echo signal acquisition and decease their amount. To solve this problem in the theory of multichannel communication, when the transmission channels have a common frequency band but different code modulation, a code division multiple access technology has been developed, which is called code division multiple access (CDMA) [43]. For its implementation, all elements of the antenna array are simultaneously excited, each with its unique probing pulse $s_{n}(t)$, and echoes are simultaneously received by all elements of the antenna array. Schematically, echoes measured in CDMA mode $p_{m}(t)$ are shown in Figure 16 on the right and they can be represented by the expression:

(17) $$p_{m}(t)=\sum_{n=1}^{N_{e}}p_{n,m}(t).$$

The first way to use this approach is to decode the echo signals $p_{m}(t)$ so that the *m*-th subscriber can make an evaluation of the echo signal ${\tilde{p}}_{n,m}(t)$ from the subscriber with the number *n*. For effective decoding of echo signals, the correlation function $R_{n,m}(\tau )$ of a set of coding signals $s_{n}(t)$ intended for excitation of elements of the antenna array must have the following property:

(18) $$R_{n,m}(\tau )=\int_{-\infty }^{\infty }s_{n}(t)s_{m}(t+\tau )dt=\delta_{n,m}\delta (\tau ),\;n,m=1,2,\ldots ,N_{e}.$$

where $\delta_{n,m}$ is the Kronecker symbol. The set of signals having the property (18) is called orthogonal. Signals for practical use with this property are not known, but several sets of coding signals *s_n_*(*t*) have been developed, which to some extent approach the ideal set with the property (18). Sets of such signals are called pseudo-orthogonal. These include phase-manipulated signals encoded by a sequence of length $N_{c}$. The law of change of the chip phase can be determined by the Kasami codes [44], Gold [45], de Brain [46], in which the phase of each chip can take values 0 or 180 degrees. It is possible to use Zadov–Chu sequences with arbitrary phase for each chip [47]. A set of sequences with a random phase can also be considered as pseudo-orthogonal. Each chip can determine the phase of one period at the carrier frequency $f_{c}$, but the option of random frequency measurement $f_{c}+\delta f$ from chip to chip is possible. For the emitting of such signals to the arbitrary pulse generator of the flaw detector, higher requirements are imposed, in comparison with the usual shock excitation generator.

The first way to restore the image is as follows. After selecting a set of code signals ${\left\{s_{n}(t)\right\}}_{n=1}^{N_{k}}$, their simultaneous pulsing and reception in one cycle, the measured echoes $p_{m}(t)$ must be decoded to evaluate the signals *p*(*t*), as if measurements were made in the FMC mode. By decoded echoes ${\tilde{p}}_{n,m}(t)$ using the C–SAFT method, one can restore the image (arrow and rectangle with a yellow fill in Figure 16). In Figure 16 the decoding procedure is likened to the passage of a beam of light through a prism. If we use the color analogy in Figure 16, the decoding with the correct selection of signals ${\left\{s_{n}(t)\right\}}_{n=1}^{N_{k}}$ will allow the signals $p_{n,m}(t)$ from the total set $p_{m}(t)$ to be “colored” only in one color. Considering the circumstance that pulses of “different colors” can be very close to each other, the algorithm of decoding should provide a high resolution in time domain.

A common method of decoding complex signals $p_{m}(t)$ is matched filtering with a code signal $s_{n}(t)$. Given that matched filtering in the time domain is equivalent to convolution [43], the decoding operation can be written as:

(19) $${\tilde{p}}_{n,m}(t)=\int_{-\infty }^{\infty }p_{m}(\tau )s_{n}(\tau -t)d\tau .$$

This algorithm for compression of complex signals has a high speed and allows one to obtain images with a frequency of more than 10 Hz, but it does not allow to obtain a low level of interchannel noise and achieve the effect of super-resolution. For decoding of echoes by Equation (19), the level of interchannel noise, i.e., the average value of the cross-correlation function of signals in the set ${\left\{s_{n}(t)\right\}}_{n=1}^{N_{k}}$, can be estimated as the value of ${\left(N_{c}\right)}^{-\frac{1}{2}}$.

A more complex method of decoding simple or complex signals is based on the use of the maximum entropy method [37] and was considered in the section Processing of echo signals presented in a complex form. To obtain the estimation of echo signals by the ME method by measured ${\tilde{p}}_{n,m}(t)$ for each $s_{n}(t)$ from a set of coding signals ${\left\{s_{n}(t)\right\}}_{n=1}^{N_{k}}$, it is necessary to form a matrix G. The time of decoding of echoes $p_{m}(t)$ by the ME method is much longer than by the Formula (19), which will lead to decreasing the frequency of image formation. But for an automated system with post-processing, such a speed limit is not essential.

The second way to obtain the image is to get the image of the reflectors immediately using the ME method without decoding $p_{m}(t)$, as described in section Restoring the image of reflectors. This method in Figure 16 corresponds to an arrow and a rectangle with a green fill.

The efficiency of the algorithms for decoding echoes depends on the type of reflectors being reconstructed and their quantity, since this depends on how close to each other the echoes are located. That is, the quality of the decoding of echoes from a single point reflector can be very good [48], but when processing echoes from many reflectors, the reconstructed image may end up with an unacceptably high level of inter-channel interference. Ideally, the use of CDMA technology will allow to measure for an antenna array of 128 elements only in one cycle instead of 128, and decrease the number of echoes from 16,348 to 128.

The model experiment was carried out on the sample described in Section 4 with drilled holes in the rescattering test, but using the electronic unit of the AUGUR-ART system, which allows pulsing complex signals. As a set of complex signals, 32 ($N_{k}=N_{e}=32$) phase-manipulated signals according to the Kasami code were used with length $N_{c}=15$. Each period had a frequency randomly distributed in the [5 ± 2] MHz band. To acquire echoes, an antenna array was used (5 MHz, 32 elements, crystal size 0.9 × 10 mm, 0.1 mm pitch) on a rexolite prism with a slope angle of 35 degrees.

**Figure 17:**
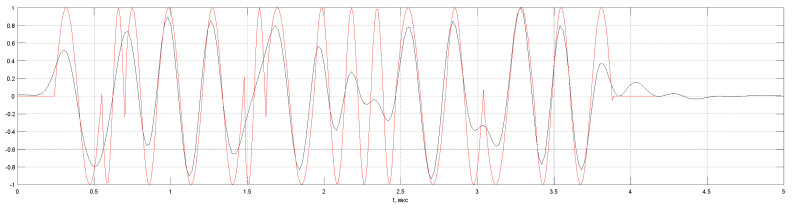
An example prepared for emitting the complex probing signal (the graph in red) and the measured echo signal (the graph in black).

Figure 17 shows an example of one complex probing signal (red graph) prepared for study and emitted and received by the equipment used (black graph). One can see that the limited bandwidth of the antenna array distorts the ideal shape of the probing signal: in the received echo signal, sharp phase jumps are actually lost when the chip changes and the signal amplitude changes from chip to chip.

**Figure 18:**
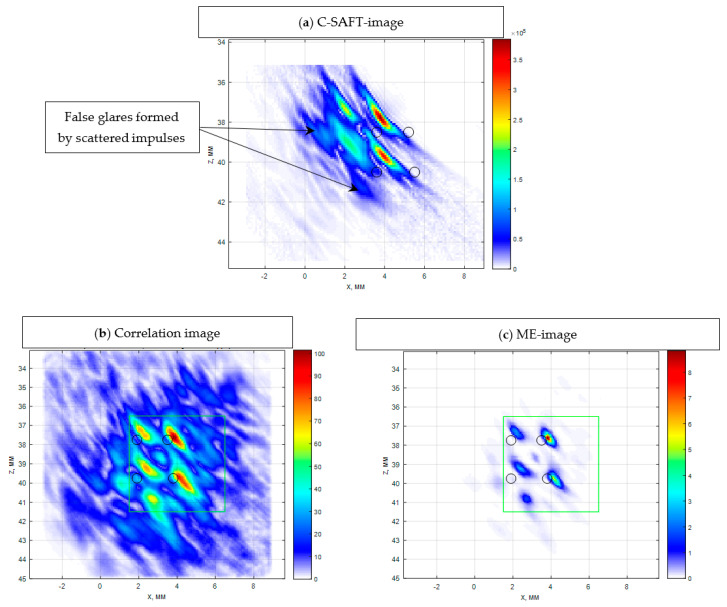
The image recovered by the method from the set of echoes measured by the emission of a simple signal in the FMC mode (**a**), by the correlation method with decoding by the matched filtering (**b**) and the ME method without decoding (**c**).

Figure 18a shows the image of the four side drilled holes reconstructed by the C–SAFT method in the set of echoes measured during the emission of a simple signal in the FMC mode. There are three indications corresponding to the boundaries of the three holes, which are accurately resolved since the reconstruction made for transverse wave. But apart from them, the pulses scattered between the holes formed several false indications, which do not allow us to determine not just the type of reflectors, but also their quantity. The signal-to-noise ratio of the image can be estimated as 28 dB. Figure 18b shows the image reconstructed by the correlation method according to Equation (6) during decoding by matched filtering. Due to the high level of inter-channel interference, the image quality due to a low signal-to-noise ratio of 12 dB has an unacceptably low quality. Figure 18c shows the result of the reconstruction of the image of the reflectors with 32 echoes $p_{m}(t)$ by the ME method according to Equation (8) (α=25.0, $\mu =10^{-5}$). The longitudinal and frontal resolution of the image increased more than twofold in comparison with the image of Figure 18a. On the ME image, the indications of the four holes are clearly visible, and one false indication with a commensurate amplitude. It should be recalled that the acquisition time of echoes $p_{m}(t)$ is in $N_{e}$ times shorter than the measurement time in FMC mode. The data set of the measured echo signals is as much smaller.

## 7. Conclusions

The use of the maximum entropy method allows one to improve the quality of the reconstructed image of the reflectors and thereby increase the reliability of ultrasonic non-destructive testing. The use of the ME method in the model experiments increased the resolution of the image by a factor of three in comparison with the linear reconstruction methods. This made it possible to more accurately determine the type and number of reflectors.

Compression of complex signals by the ME method made it possible to increase the signal-to-noise ratio more than by 20 dB for the imaging in materials with a high attenuation, which made it possible to detect reflectors at a great depth with superresolution. The ME method is equivalent to reducing the duration of echo signals length, and this should reduce the level of structural noise. In the model experiment, it was possible to reduce the level of structural noise of the reflector image by 12 dB.

Since when processing echoes measured in TOFD mode, the shape of the echoes depends on the depth of the reflectors, a variant of the ME method, taking this into account, was proposed. In a numerical experiment, the effectiveness of this approach is shown.

The possibility of using the ME method for obtaining a 2.5D-image of the reflectors in a reference block made from 800 mm diameter pipe. Application of the ME method together with CDMA technology allowed to reduce the echoes acquisition time by FMC and obtain an image with superresolution. In the model experiment it was possible to increase the rate of echo signal registration for the 32-element elemental array by 32 times. With the increase in the number of elements of the antenna array, the proposed approach further increases the rate of registration of echoes. This can be very important for ultrasound medical diagnosis.
